# The pro-environmental behavioral intention of villagers in rural tourist destinations under China’s environmental remediation policy

**DOI:** 10.1038/s41598-023-39998-3

**Published:** 2023-08-25

**Authors:** Hongjiu Tang, Di Hu, Yuelin Long, Yuqi Zhao

**Affiliations:** 1 College of Landscape Architecture and Art Design, Hunan Agricultural University, Changsha, China; 2 College of Resources and Environment, Hunan Agricultural University, Changsha, China

**Keywords:** Human behaviour, Psychology and behaviour

## Abstract

This study examined villagers’ intention of pro-environmental behavior while supporting the Landcare Policy in China. The research team conducted field surveys of villagers from four famous scenic spots of Cili, which is near the world natural heritage site of the Zhangjiajie natural landscape core area. This area has developed rural tourism, many local villagers rely on tourism to obtain their livelihood income. However, the area is now affected by the environmental remediation policy called Landcare Policy. Cultivated land near the tourist area needs to be repaired, which affects the tourism income of some local villagers. Therefore, local villagers are facing a contradiction between tourism development and environmental protection. The study chose the change in local villagers’ pro-environmental intention as the research content. Then we adopted an empirically validated norm activation model (NAM) from Schwartz, and merged the NAM with the expectancy theory of Vroom, based on 511 valid responses from the field questionnaire surveys, we aimed to develop a theoretical framework for researchers to understand the change in villagers' pro-environmental behaviors, concerning the balance between rural tourism livelihood benefits and environmental remediation behavior. Structural equation modeling was conducted for each index of the responses, the findings showed that the merged model had 76.46% better predictive accuracy of villagers’ pro-environmental intentions than applying Schwartz’s NAM independently. This study found that the motivational force of this new theory significantly influences environmental personal norms due to the joint impact of valence, instrumentality, and expectancy. Villagers with a positive pro-environmental behavior intention expect good tourism benefits and environmental living conditions under the impact of the Landcare policy in rural tourism destinations near the famous natural heritage site.

## Introduction

The rapid development of tourism can produce positive external economic benefits for surrounding villagers, especially in remote and underdeveloped rural areas^1^. Therefore, many villagers around famous scenic spots actively engage in tourism livelihood activities. There are also many mineral resources in the vicinity of some remote scenic spots, so there are many mineral processing and production enterprises in the past, such as nickel molybdenum mining. The underdeveloped technology of local mineral production coupled with the lack of attention to environmental protection by managers and the high cost of waste treatment have led to the disorderly discharge of polluted water, which contains a large amount of heavy metals that seriously exceed the heavy metal content of local cultivated lands. This affects the quality of local agricultural products, villagers’ health, and tourists’ safety. The Chinese government proposed the cultivated Land Remediation Policy to solve this pollution problem. On May 20, 2016, the pilot program for implementing a system of crop rotation and Landcare for cultivated land was approved^2^.

The Landcare reserve policy (called Landcare Policy, a kind of environmental remediation policy) under the background of heavy metal content exceeding the standard in part of rural areas, and these local management government began to implement a mandatory cultivated land remediation policy in areas of rural areas with excessive heavy metal content in land^1^. To achieve this goal, it is necessary to establish a protective isolation zone for heavy metal pollution diffusion on polluted cultivated land and repair cultivated land that has been polluted by heavy metals using physical, chemical, biological, and other means^3^. This will force some villagers who engage in rural tourism for their livelihood to take time to cooperate with the government's cultivated land remediation activities. At the same time, their rural tourism businesses will be restricted by the government, and these villagers will receive fewer land care subsidies from the government due to the reduction of local tax revenues. In fact, their tourism and agricultural livelihoods will be significantly reduced due to the completion of the government's Landcare policy. These villagers engaged in rural tourism who are affected by the Landcare Policy will face a contradictory problem of fulfilling their social responsibility for environmental protection in the face of a significant decrease in tourism and agricultural livelihoods income. What factors affect their pro-environmental behavior, and how will it change?

Cili County is a remote and underdeveloped county in the northwest of Hunan Province in China. It is located in the most impoverished area in China, the Wuling Mountain area. However, it is close to a famous world natural heritage site (Zhangjiajie Wulingyuan-Tianmen Mountain Scenic Area), which is a world-renowned high-quality natural sightseeing and leisure spot. A large number of foreign tourists bring employment opportunities to the villages around this scenic spot. Many local villagers use local agricultural products and tourism service activities to engage in tourism livelihood activities^4^. Cili County in the past due to the mining of nickel ore, molybdenum ore (the county nickel molybdenum mining and processing enterprises all stop production in 2013), many heavy metals in a part of rural cultivated land exceed the standard^5^. Due to the mandatory constraints of China's Landcare Policy since 2016, many local villagers who engage in tourism livelihoods must cooperate with the government's cultivated land remediation work^6^.

Experts, practitioners, and policy-makers have often disagreed about the connection between the growth of tourism and environmental protection. The lack of effective guidance on the balance and management between environmental protection and tourism development is detrimental to the expectations of sustainable social development in local urban and rural areas. To make sustainable use of rural tourism resources, it is necessary to study the influence of villagers’ behavioral intentions and identify ways to improve their enthusiasm for environmental protection. Mbaiwa and Stronza^7^ found that if villagers derive benefits from rural tourism resources, they will feel obliged to use local tourism resources sustainably, and they will show more positive pro-environmental behavior. Villagers' pro-environmental behaviors are also influenced by their awareness of the natural environment^8^. Therefore, the aim of this study is to examine a scientific theoretical framework based on various theories and contexts related to the factors that affect environmental protection and to explain the influence of pro-environmental intention on the ecological restoration of tourist destinations under China’s Landcare Policy.

The research on rural tourism tends to focus on the aggregate effect of different public behaviors. Existing research results suggest that most people show more responsibility for environmental protection at tourist attractions than at home^9^. This study focuses on pro-environmental behavior from a broader perspective in which pro-environmental behaviors are not necessarily related to specific tourism products or services. The pro-environmental behaviors of villagers in the ecological restoration area can make a positive contribution to the environmental improvement of rural tourism destinations. Therefore, villagers’ pro-environmental behaviors provide important public protection for the sustainable development of rural tourism^1^.

In early studies of pro-environmental behavior, social theoretical models based on awareness, attitudes, and morality related to environmental protection were widely used by researchers. Most public campaigns therefore attempted to raise public awareness of rural environmental damage issues. Unfortunately, scholars found that this approach was ineffective. The study of ecologically friendly behavior is mainly conducted from two aspects: altruism and self-interest. Tourism researchers who think that environmentally responsible behavior has prosocial motivations usually adopt theories based on ethics, such as Schwartz's norm activation model (NAM)^10^ or Stern's value-belief-norm theory (VBN)^11^. In contrast, some researchers believe that environmentally friendly behaviors are driven by people’s interests and are based on expectations of future benefits that encourage villagers to conduct environmental protection under the policy^12^. The theory of self-interest is based on the premise that behaviors are driven by desirable outcomes or ideal rewards. Handrito notes that the best approach is to combine the two views^13^. Keshavarz and Karami found an attitude-action gap among villagers of different ages. Local villagers are more directly related to the agricultural production of cultivated land, so they have relatively strong environmental awareness of the excessive heavy metal content in cultivated land^3^. However, migrant villagers are less inclined to take positive actions than local villagers. Generally, local villagers, specifically those who believe that they belong to a certain place, will show a more active attitude toward environmental protection.

To promote the country's cultivated land remediation policy, it is important to study the changes in the pro-environmental behavior of villagers who have lost their livelihood income after being constrained by environmental protection policies, to improve environmental protection policies and measures around scenic spots and to improve the long-term livelihood income and social welfare of local villagers. Theoretically, a significant and concerned attitude toward environmentally friendly behaviors is necessary. An advantage of adjusting the sample population is that this study pays close attention to these changes in the theoretical framework, while samples composed of homogeneous members will help to reduce potential model errors influenced by other mediators. Peng noted that in general, research on the evolution and characteristics of pro-environmental behavior in tourist destinations tends to choose local villagers on a large scale^9^. The study samples were designed specifically to include local villagers who are engaged in tourism to represent the villagers affected by the Landcare Policy in the ecologically restorative tourist destination. This study attempts to fill this gap by merging Schwartz’s norm activation model and Vroom's expectancy theory to investigate the pro-environmental behavioral intentions of local villagers under the impact of the cultivated land remediation policy in China.

## Literature review

### Pro-environmental behaviors of villagers

The phrase “pro-environmental behavior” refers to a broad variety of actions and may be interchanged with other terms, such as ecologically friendly behavior. This study adopts Schwartz’s definition of pro-environmental behavior, which includes any behavior that protects the environment in daily work, in the natural environment, or in the outdoor environment to minimize the negative impact of human activities on the environment^14^. These daily agricultural practices include activities such as agricultural fertilization, agricultural pest control, agricultural waste treatment, physical degradation of heavy metals in cultivated land, and the use of quicklime to reduce the pH of the soil. This study measures the common pro-environmental behaviors of villagers in the process of implementing the agricultural Landcare Policy in an ecologically restorative tourist destination. The final construct, which measures behavioral intention, is adopted from broadly accepted prior research^4, 14–16^. These include giving priority to accepting the guidance of agricultural experts on Landcare activities, sacrificing rest time to promote soil remediation technology, persuading others to protect the ecological environment, spending money to improve tourism service facilities, and learning relevant environmental protective skills and knowledge^6^.

Previous studies have found that gender, age, social background, income level, education, and other factors can affect a person's pro-environmental attitude and behavior^4^. The theory used in this study measures environmental behavioral intentions because they are found to be important predictors of awareness and norms^14^. Agliardi and Agliardi^15^ noted that some respondents linked environmental protection behavior to time costs and higher spending constraints, although most of them showed good environmental awareness and positive environmental attitudes. Olya and Akhshik^16^ also considered hurdles to pro-environmental conduct, such as financial costs, emotional input, and awareness of responsibility. In view of the background of this study, financial cost and responsibility may explain the environmental protection intention of villagers in ecological restoration areas. The purchase of agricultural governance information and other behaviors may be less of a concern for villagers. Under the Landcare Policy, the economic benefits brought by their pro-environmental behavior will be given priority.

### Norm activation model

Schwartz^10^ originally developed the norm activation model (NAM), which involves altruistic behavior. "Personal norms" are the core element of this model. The public's positive attitude is recognized as one of the most powerful forces in resolving various societal concerns. The challenge of public environmental protection is a large-scale issue that requires social movements^17^. The public movement of environmentalism is a widespread behavioral change process that enterprises, organizations, and individuals must make to realize the goal of diminishing the adverse impact of human beings on environmental change^18^.

Feelings of environmental responsibility activate personal norms, and these norms induce the intention to engage in pro-environmental behavior^19^. According to the NAM theory, there are four components to pro-environmental conduct: the behavioral intention, attribution of responsibility, personal norms, and awareness of consequences^10^. Based on the four components of the model, this study analyzes and discusses daily agricultural practices and tourism activities related to rural cultivated land in rural areas. Schwartz considered four basic components relevant to an ideal cross-situational goal; these components have different importance as environmental protection behavior requirements in the daily practice of individuals or other social entities^14^.

Awareness of environmental consequences (AEC) is the first construct in the NAM framework in relation to the Landcare Policy. It refers to the awareness that the problem of environmental protection can be enhanced or diminished by the biosphere, people, and other species^11^. Collective opinions and judgments frequently impact or increase with environmental awareness^10^. Environmental consequences have long been a subject of concern in a variety of fields, and empirical evidence supports their role as predictors of environmental attitudes. Schwartz and Boehnke^18^ explained that the construct of awareness of consequences is complex and can be composed of many variables.

Ascription of environmental responsibility (AER) is the belief that individuals’ behavior can reduce or exacerbate the potential negative impact of consequences^20^. Montada and Kals^21^ found that the ascription of responsibility is the main contributor to villagers' willingness to support environmental protective policy. When compared to other theories, the link between all the variables of the NAM frequently generates strong statistical findings, but the predictive capacity has limitations. Han^22^ found that the ascription of responsibility in the NAM theory had unsatisfactory predictive power in the rational behavior of some public activities.

Environmental personal norms (EPN) are social rules that guide how individuals should comply and are restricted by the ascription of responsibility^14^. Loureiro et al.^4^ subdivided personal motivation according to personal norms. The findings highlight the potential for the segmentation and measurement of pro-environmental behavior change, they suggest that personal norms are sensitive to the use of cultural interpretation^4^. At the end of this cause‒effect chain is the last construct of the study, which examines the intention of pro-environmental behavior (IPB). Overall, the three influence constructs of Schwartz's NAM are awareness of consequences, attribution of responsibility, and personal norms, which are believed to successfully predict personal intention regarding conduct^10^. Many scholars have experimentally confirmed the framework^20, 23^. Definitions of the main terms are summarized in Table 1.

**Table 1:** Definitions of each construct.

Variable	Definition
Awareness of environmental consequences (AEC)	“Awareness that the current state of the environment will pose a real threat to other species, the natural biosphere, and human beings”. or “Awareness that the current situation of the ecological environment will threaten the values of individuals”^10^
Ascription of environmental responsibility (AER)	“Environmental actions initiated by individuals or organizations could avert these long-term or short-term negative consequences”^24^
Environmental personal norms (EPN)	“The feelings of a particular environmental action that is morally or ethically considered to be obliged to do or not to do”^10^
Intention of pro-environmental behavior (IPB)	“Any actions that protect the environment or minimize the negative impacts of human activities on the environmental remediation activities in either general daily practice or specific outdoor settings under the impact of the Landcare Policy”^14^
Valence (Val)	“The value the individual personally places on praise or rewards”^12^
Instrumentality (Ins)	“The perceived probability that good performance can result in desired outcomes”^25^
Expectancy (Exp)	“The perceived probability of doing one’s best to achieve good performance or results”^12^

Because of these analyses and evidence, the causal chain of the NAM can be constructed as follows: AEC → AER → EPN → IPB. The following hypotheses are proposed (H1–H3):

- *H1* Awareness of environmental consequences has a positive effect on the ascription of environmental responsibility.
- *H2* The ascription of environmental responsibility has a positive effect on environmental personal norms.
- *H3* Environmental personal norms have a positive effect on villagers' intention of pro-environmental behavior while supporting the Landcare Policy.

### Environmental policy and expectancy theory

The classical expectancy theory was proposed by Vroom^12^. His viewpoint is based on studies in industrial and organizational psychology. Many academics in the tourism field utilize this framework to capture tourism product suppliers' motivations for improving a destination's image, community harmony and service attitude. A search of the literature showed that there was no clear application of expectancy theory in the analysis of pro-environmental behavior using the NAM theory. However, after reviewing the NAM theory and other relevant theories, the authors found that the two theories might be synergized. The theory of expectancy is used to describe how people make decisions regarding different behavioral choices^24^. Three separate constructs are included in the framework: valence, instrumentality and expectancy. The motivations for certain activities are determined by these three conceptions. The first is valence, which defines the driving force or appeal of a given result. Expectancy is the second component of expectancy theory, which is the perception that a certain amount of performance will lead to the desired result. The third is instrumentality; the greater the amount of work needed, the greater the possibility that the desired objectives will be accomplished. People believe that actions lead to positive expectations. In the study of Keshavarz and Karami^26^, this theory was applied to predict the extent to which individuals support their environmental protective beliefs in relation to the effectiveness of individuals’ performance as well as the extent to which individuals’ work is affected by the effort invested under the impact of environmental policy and effectiveness with regard to the effort ultimately ensuring the expected results. Therefore, the framework is expressed as follows:

$${\text{Motivational}}\;{\text{force}}\;{\text{of}}\;{\text{villagers'}}\;{\text{intention}} = {\text{Valence}} \times {\text{ Instrumentality}} \times {\text{Expectancy}}$$

In this expression, the three components are measured separately and collectively constitute the motivational force. However, the study suggests that these three components should be treated within a chain of causal influences, leading to a motivating intention that is conducive to pro-environmental behavior when supporting the Landcare Policy. Many explanations have been presented since the expectancy hypothesis was proposed. The lack of clarity in defining the relationships between various motivational force models has led academics to examine how one affects the others^27^. Additionally, many studies examine the concept of causal relationships. Therefore, the preceding causal chain is depicted as follows:

$${\text{Valence}} \to {\text{Instrumentality}} \to {\text{Expectancy}} \to {\text{Motivational}}\;{\text{ force}}$$

In this case, valence is at the beginning of this chain, which appears as a villager's value for a particular outcome. The goal includes the desire for things such as living in a rural area free of heavy metal pollution. This leads villagers to have the expectation that the more effort they make to reduce heavy metal pollution, the better the local tourism industry will be. Individual contributions may be small, but they are still effective overall. To underline the importance of effort, valence is expected to have a positive impact on specific outcomes. It is believed that taking action will result in the expected instrumentality and that instrumentality will have a beneficial influence on an individual’s level of expectation. Expectancy can affect environmental behavior when supporting the Landcare Policy in China. From the discussion of expectancy theory, three assumptions are proposed:

- *H4* Valence has a positive effect on instrumentality.
- *H5* Instrumentality has a positive effect on expectancy.
- *H6* Expectancy has a positive effect on villagers' intention to behave pro-environmentally.

### The merger of the two theoretical frameworks

Discussions of the individual construct of sociopsychology are more predictive of pro-environmental behavior than discussions focusing on the demographic background of the sociopopulation^28^. Variables such as awareness, responsibility, norms, and behavioral intention regarding the environment are empirically related to personal feelings and ways of thinking and ultimately affect behaviors related to environmental problems^29^. The theory has been clearly confirmed, and its effectiveness has been tested^19^. In addition, compared to other theories, the prosocial construct of the NAM model has led researchers to agree that it has superior predictive power. However, the NAM has been studied along with other well-known attitude theories, such as Stern’s value-belief-norm theory^30^. Van and Meertens^31^ suggested that the combination of pro-social and rational choice theory can produce higher predictive ability. Park and Ha^32^ combined the NAM and planned behavior theory into a single theoretical framework. Their findings support the suggestion of Van Vugt et al.^31^ that the model shows better precision for consumer recycling behavior than the independent NAM framework. The successful application of the merged model in interpreting an individual’s environmental behavior can demonstrate the validity of the NAM. In contrast, adopting the expectancy theoretical framework helps to improve the performance of separate NAMs. Although expectancy theory is a research framework that has been widely accepted worldwide, there are still some criticisms from researchers^33^. A prominent criticism of this theory is the lack of consideration of social impact^34^. By fusing expectations theory with the NAM model, it is assumed that this limitation will be reduced. The NAM originated from a theory that studies social movements. These measurement items involve individual awareness, responsibility, and personal norms about behavioral intentions for environmental protection, which affect both human society and the local environment. The important view is that expectations of a better living environment and income growth may affect one's eagerness to help protect the local environment^35^. Yuan et al.^36^ suggested that personal norms are usually formed by social interaction, but the final decision is made by one’s expectations. In other words, valence may affect the amount of an individual's actual effort and ultimately affect the ascription of environmental responsibility. Individual contributions might be minor but cumulatively impactful, highlighting the importance of effort (instrumentality). It is predicted that instrumentality has a positive influence on environmental personal norms. Expectancy theory measures a narrower range of variables than the theory of NAM, and the NAM does not need to consider the frequency of actions. Improving villagers' intentions to support the Landcare Policy requires repeated efforts rather than one-off actions. The effective impact of expectancy is essential to promote broad improvements in environmental personal norms. Based on these discussions, the links between the three theories are hypothesized as follows:

- *H7* Valence positively affects the ascription of environmental responsibility.
- *H8* Instrumentality has a positive effect on environmental personal norms.
- *H9* Expectancy affects environmental personal norms.

**Figure 1:**
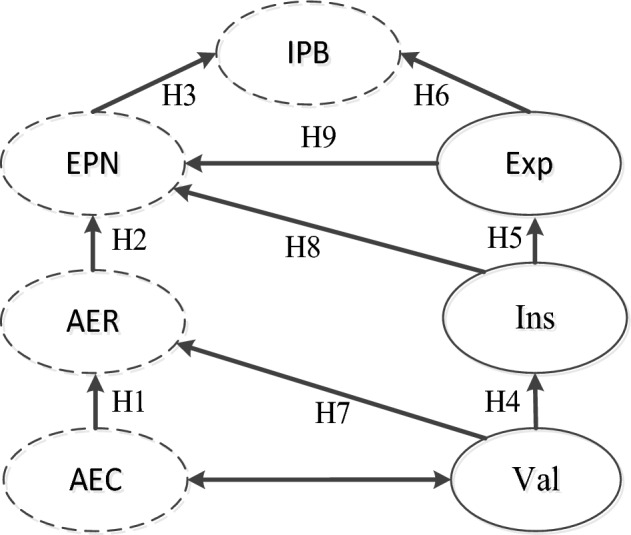
The proposed research model. *Note*: the NAM is denoted by constructions with dashed outlines. (H1 → H2 → H3). The expectancy model is represented by solid-lined constructions. (H4 → H5 → H6).

The proposed theoretical model includes a total of 7 constructs and 9 hypotheses. The interactive relationships between the seven constructs are illustrated in Fig. 1.

## Methodology

### Statement

## A statement to confirm that all methods were carried out in accordance with relevant guidelines and regulations.

Authors reporting experiments on humans and/or the use of human tissue samples must confirm that all experiments were performed in accordance with relevant guidelines and regulations.

This study guarantees respect for the individual will of each questionnaire participant and their right to know the research content. The questionnaire was conducted with the explicit consent of each respondent. We ensured that each researcher was clear about the purpose, content, and data use of the questionnaire. Our study data are based on first-hand data obtained from an anonymous survey of the respondents. Before conducting the questionnaire survey on potential research subjects, our investigators formally explained to each tourist face-to-face after project training that this survey was anonymous.

## A statement to confirm that all experimental protocols were approved by a named institutional and/or licensing committee.

This study and all experimental protocols were approved by the *Biomedical Research Ethics Committee of Hunan Agricultural University* (Institute of Research) and the *Ethical Approval Section of Hunan Agricultural University No. 106, 2022* (licensing committee).

Our research activities were carried out in accordance with the requirements of the project application and in line with the ethical requirements of the study. The work of the research team was regularly inspected by the ethical approval agency. The data obtained are about participants' psychological attitudes and behavioral intentions regarding ecological pro-environmental behavior in tourist destination scenic spots. There was a Chinese explanation on our questionnaire that introduced the purpose of this research, the research discipline, the protection of personal privacy, and the respondents’ right to stop answering questions at any time.

## All the authors of this paper confirm that informed consent was obtained from all subjects and/or their legal guardian(s).

### Measurement instruments

In this study, a five-point Likert scale was used for all latent variables, and there were no recording problems. Scores on the questionnaire were on a scale from (1) "extremely disagree" to (5) "extremely agree". Before the final version of the questionnaire was confirmed, the research team recruited seasoned academics, environmental specialists, and students to conduct pretests and assessments. Based on their feedback, minor adjustments were made to the grammar, formatting, wording, visual construct, and psychological experience of the questionnaire. Since the sample comprised villagers engaged in tourism in Cili County, which is an ecological restorative tourist destination affected by the Landcare Policy, the final version of the questionnaire was translated into Chinese to enable rural villagers, many of whom have little education, to understand or read it. To avoid bias and personal input and to ensure successful translation, the translators had no prior knowledge of the research content. The results were in agreement with the Chinese scale version. There was a cover paragraph at the beginning of the questionnaire that summarized the research objectives and provided a brief description of the field of study. Finally, the questionnaire included demographic issues; however, sensitive personal issues and personal contact information were not involved. In order to effectively measure the pro-environmental behavioral intention of villagers around the tourist destination, this paper uses a two-step approach proposed by Cheung and Chan^37^. The first stage is to test the behavioral model’s goodness of fit, the research data were screened to check for possible violations of some proposed hypotheses. The second stage is to examine the structural model in the study, structural equation modeling was used to test the goodness-of-fit with the proposed model.

### Case selection

Cili County is close to the eastern part of the world-famous Wulingyuan-Tianmen Mountain tourist area in Zhangjiajie City, Hunan Province, China. Cili County has the honorary titles “town of hot springs in China” and “the most beautiful leisure tourism resort county in China.” Cili County is a poor county with many mountains and few roads. However, it has world-class, high-quality tourism resources. The county has world-renowned tourist attractions such as Zhangjiajie Grand Canyon, Zhangjiajie Longwang Cave, Wulei Mountain and Jiangya Hot Spring. Tourism economy and agricultural development are important sources of livelihood income for villagers^4^.

Cili County is also a county with serious heavy metal pollution. The nickel-molybdenum mining area in Cili County is one of the 138 comprehensive prevention and control areas for heavy metal pollution listed by the Ministry of Environmental Protection of China^4^. According to the results of an intensive survey of heavy metal pollution of agricultural land in Hunan Province of China in 2019, the local Landcare action involves 54 villages in 15 towns or township administrative regions, 9 enterprises or cooperatives, 2 family farms, 7 large planting households and 3349 villagers^5^. At the beginning of 2020, the local government decided to start the treatment and restoration of polluted cultivated land in Cili County, which made some villagers on cultivated land with excessive heavy metal content change their agricultural livelihood. At the same time, some tourist attractions had to make local adjustments due to their proximity to heavy metal-contaminated areas, which affected the tourism livelihood of surrounding villagers^38^. For this reason, the attitude of villagers toward environmental protection at tourist attractions that were negatively affected by heavy metal pollution changed accordingly.

### Data collection

Data were collected by the author and three undergraduates (graduating in 2023) majoring in human geography and urban planning of Hunan Agricultural University. The four investigators conducted field surveys in the *Zhangjiajie Grand Canyon, Zhangjiajie Longwang Cave, Wulei Mountain and Jiangya Hot Spring*. The research team selected villagers engaged in rural tourism near these four famous scenic spots from November 7, 2022, to November 13, 2022. At each of these four attractions, the researchers expected to issue 160 questionnaires. The author provided training and guidance to the investigators before departure. Before receiving the questionnaire, respondents were asked if they were villagers around Cili County ‘s’ villages, whether they understood the Landcare Policy, whether they were affected by the Landcare Policy, whether they were currently involved in the tourism industry, and whether they agreed to fill out the questionnaire anonymously for academic study. The questionnaires were issued only after verbal agreement by the villagers^39^. The survey sample comprised villagers living in ecological restoration areas who engaged in tourism. Because the overall identity of the sample was relatively consistent, which provided a relatively homogeneous sampling profile for research and analysis, the collected data had high internal validity. The standard deviation of each response was calculated to remove unengaged responses if a value less than 0.5 was obtained in the review of patterns exhibiting nonengagement of the respondents. Thus, 21 responses were deleted for showing unengaged performance. The research team distributed 560 questionnaires, and the actual number of valid responses was 511. The questionnaire’s effective rate was 91.25%. For a few missing data, this paper used the series mean for replacement and then further analyzed these valid responses.

### Sample profile

Some villagers of Cili County were the research sample, and most of them regarded tourism as their main source of livelihood income. A sample summary is shown in Table 2.

**Table 2:** Demographic characteristics of valid responses.

Variable	Category	Distribution (person)	Valid percentage (%)
Gender	Male	244	47.75
	Female	267	52.25
Age	Mean	32.47	
	Under 18 years old	6	1.17
	18–24	272	53.23
	25–35	101	19.77
	36–50	83	16.24
	51–60	41	8.02
	Over 60 years old	8	1.57
Education	Primary school and below	4	0.78
	Junior high school	16	3.13
	Senior high school or technical secondary school	61	11.94
	College degree or higher vocational education	75	14.68
	Bachelor degree	318	62.23
	Graduate degree or above	37	7.24
Job types	Full-time work in tourism	446	87.28
	Part-time work in tourism	65	12.72
Annual income (after tax) exchange rate in November 7, 2022 ($1 = ¥7.223)	Under $1661	141	27.59
	$1662–$3322	82	16.05
	$3323–$4984	82	16.05
	$4985–$8307	66	12.92
	$8308–$13,845	59	11.55
	$13,846–$27,689	48	9.39
	$27,690–$41,534	20	3.91
	Over $41,535	13	2.54

There were 511 valid study samples. Of these, 47.75% of respondents were male and 52.25% were female. Therefore, the ratio of males to females was close to 1:1, which is in line with the local village officials' judgment. The average age of the 511 valid respondents was 32.47 years old, so the respondents were mainly middle-aged and elderly. Six persons were under 18 years old, 272 were aged 18–24, 101 persons were aged 25 to 35, 83 persons were aged 36–50, 41 persons were aged 51–60, and 8 villagers were over the age of 60. The majority of respondents had a middle education level; 4 villagers completed primary school and below; 16 villagers were junior high school degrees; 61 villagers were senior high school or technical secondary school graduates; 75 villagers were college graduates or graduates of higher vocational education; 318 villagers had bachelor’s degrees; and 37 villagers had graduate degrees or above. Furthermore, 87.28% of respondents considered tourism their full-time work, and only 12.72% of respondents considered it part-time work. With regard to the annual income (after tax) obtained from local tourism, 27.59% of villagers made less than $1661 (12,000 yuan), 16.05% of villagers made between $1662 and $3322 (12,001 to 24,000 yuan), 16.05% of villagers made $3323 to $4984 (24,001 to 36,000 yuan), 12.92% of villagers made $4985 to $8307 (36,001 to 60,000 yuan), 11.55% of villagers made $8308 to $13,845 (60,001 to 100,000 yuan), 9.39% of villagers made $13,846 to $27,689 (100,001 to 200,000 yuan), 3.91% of villagers made $27,690 to $41,534 (200,001 to 300,000 yuan), and 2.54% of villagers had annual income over $41,535 (over 300,000 yuan) (the exchange rate of RMB to USD was 7.223 yuan to 1 dollar on November 7, 2022).

It can be inferred that this income was from local tourism after-tax income distribution, which is relatively optimistic compared with the income level of other local villagers who were not engaged in tourism. Under the impact of the Landcare Policy, good income from rural tourism will enhance villagers' positive expectations of their future life, which will also encourage the villagers to engage in positive pro-environmental behavior. It can be inferred that the sustainable development of rural tourism is closely related to villagers' intention of pro-environmental behavior. The ratio of effective sample responses to 25 observational items was more than five times, and the number of responses was more than 500 samples, which can significantly reduce the risk of the nonnormal distribution of samples^38^. Therefore, the 511 responses covered the main attributes of the samples, and the data distribution was in line with the actual situation.

## Results

### Confirmatory factor analysis

Testing the behavioral model’s goodness of fit is the first stage of a two-step approach proposed by Cheung and Chan^37^. Before the CFA, the research data were screened to check for possible violations of the 9 proposed hypotheses. The test showed no apparent violation of the assumptions. The value of skewness was between − 1 and 1, and the measurement items did not exceed this value range. They were all five-point Likert items and therefore were not considered to violate assumptions. The kurtosis values of the measurement items were all below the recommended threshold of 3. Further examination revealed no violations in homoscedasticity, linearity, and multivariate normality.

The CFA factor analysis method is the maximum likelihood method, and the tested results showed that the proposed model had goodness of fit (χ^2^ = 451.966, *df* = 204, *p* < 0.001, χ^2^/*df* = 2.216, IFI = 0.954, TLI = 0.931, NFI = 0.919, PGFI = 0.586, RMSEA = 0.049, CFI = 0.953, GFI = 0.934). All the indices showed that the measurement model had good acceptability according to Gholami et al.^40^. The CFI scores of the 511 valid samples were accepted because they were all above 0.90 with 25 observational items. Each latent variable involved multiple measurement items. To verify the internal consistency, a composite reliability test was conducted between different latent variables. The test scores showed that the values of each indicator were larger than most scholars' accepted minimum threshold of 0.70, and their scores ranged between 0.778 and 0.845. In Table 3, the validity test of the construct is presented. The convergence validity of different latent variables shows that the average variance extraction (AVE) score was between 0.502 and 0.656, which was larger than the generally accepted minimum requirement of 0.5^40^. Finally, the AVE scores are larger than the correlation between the constructs, providing evidence for discriminant validity^41^.

**Table 3:** AVE, ASV, correlation, reliability, mean and standard deviation.

	AEC	AER	EPN	IPB	VAL	INS	EXP	AVE	ASV
AEC	0.803^a^	0.047^c^	0.09	0.164	0.032	0.273	0.002	0.521	0.019
AER	0.002^b^	0.778	0.785	0.415	0.642	0.248	0.034	0.546	0.211
EPN	0.008	0.616	0.818	0.442	0.614	0.298	0.035	0.601	0.214
IPB	0.027	0.172	0.195	0.826	0.496	0.32	0.361	0.552	0.146
VAL	0.001	0.412	0.377	0.246	0.806	0.261	0.235	0.511	0.193
INS	0.075	0.062	0.089	0.102	0.068	0.845	0.082	0.65	0.067
EXP	0	0.001	0.001	0.13	0.055	0.007	0.815	0.528	0.032
Mean	2.531	3.971	4.108	3.772	3.897	3.618	2.934		
SD	0.711	0.779	0.726	0.749	0.786	0.738	1.005		

Goodness-of-fit: χ^2^ = 451.966, *df* = 204, *p* < 0.001, χ^2^/*df* = 2.216, IFI = 0.954, TLI = 0.931, NFI = 0.919, PGFI = 0.586, RMSEA = 0.049, CFI = 0.953, GFI = 0.934.

*AEC* awareness of environmental consequences, *AER* ascription of environmental responsibility, *EPN* environmental personal norm, *IPB* intention of pro-environmental behavior, *Val* valence, *Ins* instrumentality, *Exp* expectancy.

^a^Composite reliability, that is, the square root of average variance extraction.

^b^Squared correlations.

^c^Correlations.

### Structural equation modeling analysis

The second stage of the two-stage approach proposed by Cheung and Chan^37^ was to examine the structural model in the study. Structural equation modeling (SEM) was used to test the goodness-of-fit with the proposed model, and the results indicated satisfactory statistics (χ^2^ = 451.966, *df* = 204, *p* < 0.001, χ^2^/*df* = 2.216, IFI = 0.954, TLI = 0.931, NFI = 0.919, PGFI = 0.586, RMSEA = 0.049, CFI = 0.953, GFI = 0.934). Based on the recommended indices by Hair^41^, the fit results of the hypothesized model were also satisfactory. However, some suggestions with respect to the modification indices from the AMOS output showed significant improvements in the proposed model. After adding one path from the awareness of environmental consequences to expectancy (D1), the goodness-of-fit statistics improved more significantly. The originality of the proposed behavioral model of villagers was minimally influenced (χ^2^ = 437.485, *df* = 203, *p* < 0.001, χ^2^/*df* = 2.155, IFI = 0.956, TLI = 0.934, NFI = 0.921, PGFI = 0.584, RMSEA = 0.048, CFI = 0.955).

For this purpose, the structural model after the path adjustment was taken as the final model for in-depth analysis and discussion in the study. The study aimed to develop a theoretical framework for researchers to explain villagers' pro-environmental intentions while supporting the Landcare Policy in ecological restorative tourist destinations. Therefore, the adjusted final model was compared with the NAM original framework (χ^2^ = 139.323, *df* = 55, *p* < 0.001, χ^2^/*df* = 2.533, IFI = 0.966, TLI = 0.944, NFI = 0.946, PGFI = 0.504, RMSEA = 0.055, CFI = 0.966). The final model demonstrated a higher goodness-of-fit than the previously proposed model (χ^2^/*df* = 2.155 vs. χ^2^/*df* = 2.216) and a superior goodness-of-fit than the original NAM (χ^2^/*df* = 2.533). The results of the chi-square test comparing the final model and the proposed model revealed that they differed significantly (△χ^2^ = 14.481, △*df* = 1, *p* < 0.01). The goodness-of-fit of the final model was also relatively better than that of the original NAM. Moreover, these two models were clearly different from one another (△χ^2^/*df* = 0.061, *p* < 0.01). Compared to the original NAM model (R^2^ = 0.412) and the proposed model (R^2^ = 0.718), the final model had a relatively high ability to predict villagers' environmental protection intentions (R^2^ = 0.727). Based on comparative results, the final model was 76.46% more accurate at predicting intention than the original NAM independently for predictions. The comparison summary of the models is displayed in Table 4.

**Table 4:** The results of the structural model comparisons.

Goodness-of-fit	NAM	Proposed model	Final model
χ^2^	139.323	451.966	437.485
*df*	55	204	203
χ^2^/*df*	2.533	2.216	2.155
RMSEA	0.055	0.049	0.048
CFI	0.966	0.953	0.955
IFI	0.966	0.954	0.956
TLI	0.944	0.931	0.934
NFI	0.946	0.919	0.921
PGFI	0.504	0.586	0.584
IPB’ R^2^ (Adjusted)	0.412	0.718	0.727

Chi-square difference test between the final model and the proposed model:

△χ^2^/*df* = 0.061, △*df* = 1, *p* < 0.01. Chi-square difference test between the final model and NAM theory: △χ^2^/*df* = 0.378, △*df* = 1, *p* < 0.01.

*IPB* = Intention of pro-environmental behavior while supporting the Landcare Policy.

The relationships among different constructs were examined as hypothesized. From the analysis of structural equation modeling, an additional significant path was discovered. Table 5 summarizes these findings. Awareness of environmental consequences was found to be significantly related to the ascription of environmental responsibility. Environmental personal norms and expectations were shown to be substantially correlated with the ascription of environmental responsibility. Valence was found to be significantly related to expectancy. Expectancy was found to be significantly related to environmental personal norms. Expectancy and environmental personal norms were found to be significantly related to the intention of pro-environmental behavior while supporting the Landcare Policy. Therefore, Hypotheses 2, and 3 were supported, Hypotheses 1 was rejected. As expected, the relationships among the NAM constructs were not significantly related. Hypotheses 4 and 5 were supported as expected. Hence, the two constructs involved in expectancy theory were significantly related. Finally, we need to focus on the two hypothesized relationships that link the NAM framework to expectancy theory (H6 and H7). While Hypothesis H7 displayed significant relationships and was supported, Hypothesis H6 was found to be insignificantly related. Therefore, Hypothesis H6 was rejected.

**Table 5:** Standardized parameter estimates of different influence paths.

	Standardized estimate	t value	Hypothesis
H1: AEC → AER	0.005	0.121	Rejected
H2: AER → EPN	0.925	13.721*	Supported
H3: EPN → IPB	0.654	7.838*	Supported
H4: Val → Ins	0.252	4.082*	Supported
H5: Ins → Exp	0.964	2.913*	Supported
H6: Exp → IPB	0.476	4.514*	Supported
H7: Val → AER	0.822	12.531*	Supported
H8: Ins → EPN	0.109	3.407*	Supported
H9: Exp → EPN	0.012	0.150	Rejected
D1: AEC → Exp	0.378	2.340*	Discovered
Standardized total impact on intention of pro-environmental behavior	AEC = 0.186, AER = 0.605, EPN = 0.654, Val = 0.633, Ins = 0.538, Exp = 0.484		
Total variance explained:	R^2^ of IPB = 0.727		

*AEC* awareness of environmental consequences, *AER* ascription of environmental responsibility, *EPN* environmental personal norm, *IPB* intention of pro-environmental behavior while supporting the landcare policy, *Val* valence, *Ins* instrumentality, *Exp* expectancy.

*p < 0.01.

**Figure 2:**
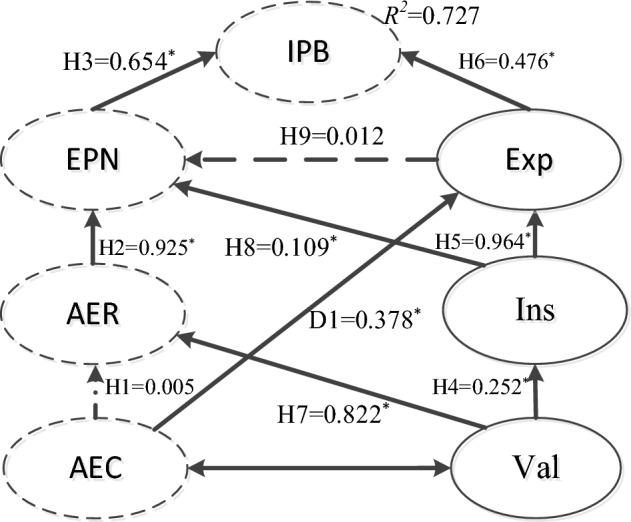
Results of the final model about the structural equation modeling. *Note*: *p < 0.01, constructs with dashed lines indicate that the hypothesis is rejected.

As mentioned above, the modification index analysis of different influence paths related to structural equation modeling indicated that the discovery of one additional relationship was not an initial hypothesis. The study made an effort to include the additional relationship in the final model, and it will be retained for future studies to further compare with other models. According to the suggested model, an additional relationship (D1) between expectancy and awareness of environmental consequences was found. In comparison to the norm activation model and the suggested model, the final model was shown to have enhanced model fit statistics due to the inclusion of the newly found routes. As a result, this research opted to retain the discovered path. Figure 2 shows the results of the final model and structural equation modeling analysis.

From the standardized total impact on the intention of pro-environmental behavior in the final model, the results indicated that environmental personal norms had the maximum total impact (0.654) on the intention of pro-environmental behavior. The level of environmental personal norms was the most important influencing factor of personal pro-environmental behavior intention. From the total variance explained in the final model, the results indicated that 72.7% of the intention of pro-environmental behavior was explained by environmental personal norms and expectancy.

The indirect impacts among constructs were assessed, and their results were as follows: except for EPN, all other factors had a positive and significant indirect impact on intentions of pro-environmental behavior, namely, awareness of environmental consequences (β = 0.186, *p* < 0.01), ascription of environmental responsibility (β = 0.605, *p* < 0.01), valence (β = 0.633, *p* < 0.01), and instrumentality (β = 0.538, *p* < 0.01). Environmental personal norms did not show a significant indirect impact on villagers’ intention of pro-environmental behavior. Additional evidence of mediation effects showed that expectancy was indirectly affected by valence (β = 0.243, *p* < 0.01). Environmental personal norms were indirectly affected by valence (β = 0.791, *p* < 0.01), awareness of environmental consequences (β = 0.009, *p* < 0.01), and instrumentality (β = 0.012, *p* < 0.01). Details of the indirect impact assessment are shown in Table 6.

**Table 6:** The assessment of indirect impact.

Indirect effect of	On					
	AEC	AER	EPN	Val	Ins	Exp
Ascription of environmental responsibility	0	–	0	0	0	0
Environmental personal norms	0.009**	0	–	0.791**	0.012**	0
Instrumentality	0	0	0	0	–	0
Expectancy	0	0	0	0.243*	0	–
Intention of pro-environmental behavior	0.186**	0.605**	–	0.633**	0.538**	0.008**

*AEC* awareness of environmental consequences, *AER* ascription of environmental responsibility, *EPN* environmental personal norm, *IPB* intention of pro-environmental behavior while supporting the landcare policy, *Val* valence, *Instrumentality* ins, *Exp* expectancy.

**p < 0.01; *p < 0.05.

## Discussion

This study attempts to combine two classical theories in the field of behavioral research. In past academic achievements, the NAM had relatively significant predictive ability for pro-environmental behavior^42^. In other contexts, expectancy theory has been used to predict pro-environmental behavior in relation to relevant stakeholders in a specific context. This study merged the NAM framework and expectancy theory to create a more accurate and robust model for predicting the pro-environmental intentions of villagers while supporting the Landcare Policy^39^.

The original NAM investigated intentions after the process of consequence awareness, responsibility attribution, and personal norms. It also analyzed daily pro-environmental behaviors and their aggregate impacts, but it could not effectively measure an individual’s “effort”. Expectancy theory has been criticized by some scholars since it was developed, although its versatility has been affirmed^43^. Expectancy theory focuses on an individual’s “effort” in the process of application^44^. For example, "the right ascription of environmental responsibility leads to villagers’ desired outcomes" and "the greater your expectations, the more positive behavioral intentions to achieve the tasks of the Landcare Policy are." Hence, the study results of these hypotheses demonstrate the rationality of the merger of these two theories.

In the various constructs of norm activation model, the causal relationship expressed by H2 and H3 is as significant as the proposed hypothesis, but H1 was rejected. The findings show that the ascription of environmental responsibility and the environmental personal norms are two important premises of pro-environmental intention. But in this integrated model, villagers’ awareness of environmental consequences does not necessarily bring their ascription of environmental responsibility to be evident. This may be the reason China’s environmental responsibility is plural and information is asymmetrical, so the ascription of environmental responsibility is not necessarily clearly judged by villagers^45^.

In the various constructs of expectancy theory, the causal relationship expressed by H4, H5, and H6 is as significant as the proposed hypothesis. The findings show that the valence (the value the villagers personally place on rewards of pro-environmental behavior) is an important premise of efforts. That is, “efforts” of pro-environmental behavior may be influenced by the desired outcomes. However, from the perspective of the NAM model, moral norms or sentiments of guilt are often predictors of pro-environmental intention. The activation of pro-environmental intentions is usually predicted by norms, ignoring the guidance of behaviors and expectations of intentions^46^.

Since two constructs were added to measure intentions from the influence factor of "effect" with respect to the expectancy theory, the predictive power of the measurement of intention was significantly improved compared with the original NAM. This supports the researchers’ emphasis on the effort’s positive effect on the predictive power of the model. As expected, when villagers feel that their pro-environmental behavior may contribute to improvements in the local environment, it has a positive effect on the behavioral intention to support the Landcare Policy and take sustainable action.

Based on the findings of Shrigley et al.^47^, who researched the gap between individuals’ attitude and action, some questionnaire respondents felt that they should persuade others to care about the ecological environment, stating, “I am willing to persuade others to protect the ecological environment of cultivated land” and suggesting their expectation of the behavioral intention to support environmental protection. This finding supports a potentially effective new approach to individual environmental protective actions under Landcare policy. It avoids positioning pro-environmental behavior and its intentions as a daily requirement or a mundane convention but promotes its outcomes (the improvement of the ecological environment) as a desirable pursuit.

The influences among the NAM's constructs showed significant correlations, consistent with Shah et al.’s^48^ and San et al.’s results^49^. As expected from the initial hypothesis, there was no significant link between valence and the ascription of environmental responsibility. The reason for this deviation from the original hypothesis could be due to other phenomena that cannot be explained or recognized in the study due to the impact of unique backgrounds and circumstances. It is not the result of the validity of the model itself. One possible reason may be that villagers are not very concerned about their social dominance, individual rewards, awareness of negative consequences, and utilitarianism. The empirical findings show that awareness of environmental consequences, ascription of environmental responsibility, and environmental personal norms are an important basis for explaining the pro-environmental behavior of individuals. Compared with other studies, the conclusions are slightly different. Kiatkawsin and Han^34^ found pro-environmental personal norms to have the highest R^2^ among the variables of the final model, whereas environmental personal norms were the largest influencing factor in this empirical study, which further illustrates how behavioral intentions are developed in two different contexts. Under the Landcare Policy for daily environmental behavior, personal norms may drive environmental intentions and corresponding actions, and these actions often involve minimal intention making. At the same time, the participative intention of behaving pro-environmentally does not happen frequently; hence, it involves a complex and deeper intention-making process. This shows that villagers in this study perceived a positive effect on the environment. The measurement scores of total impacts on behavioral intention (the final model) provide further evidence. In addition, the results suggest that personal norms exert the greatest total impact on various factors that affect environmental protective intention. This is consistent with the conclusions of Gholamzadehmir et al.^50^, who considered personal norms the most important predictor of individuals' environmental protective intentions.

## Conclusions

Structural equation modeling of each index of the responses was carried out. The findings showed that the merged model had a 76.46% better predictive accuracy for pro-environmental intentions than the original NAM independently. As we hypothesized, the relationships between these two models were found to be positively significant. Villagers who have positive intentions with respect to the environment usually expect to have good tourism income and better living conditions under the impact of the Landcare Policy. The relationship of one discovery (D1) helped us deepen the previous discussion of how personal norms play an important role in predicting villagers’ behavioral intentions. These analyses and findings show that pro-environmental behavior begins from the awareness of environmental consequences and valence with respect to the environment and that they can be influenced and then generated. Finally, testing the final construction details revealed some intriguing findings. The emergence of one pattern in practice means that villagers' environmental actions potentially involve increasing villagers’ rehabilitation cost of cultivated land and may even significantly reduce their income from tourism. The villagers' intentions and actual actions to protect the environment were less satisfactory than our initial estimations. This new finding empirically illustrated that a lack of money or reduction of previous income levels was one of the obstacles to supporting environmental behavior, whether rational or not. Given that the research sample was villagers (who were not very wealthy), obstacles to tourism livelihood were reflected in the study results. Therefore, addressing the source of environmental income and avoiding negative impacts on the tourism income of villagers is a pragmatic requirement for environmental protection under the Landcare Policy.

## Data Availability

The datasets generated and/or analyzed during the current study are available in the Harvard Dataverse^51^ **(**https://doi.org/10.7910/DVN/TKMIKM).
